# Demographic differences in diet breadth of Canada lynx during a fluctuation in prey availability

**DOI:** 10.1002/ece3.2115

**Published:** 2016-08-18

**Authors:** Christa M. Burstahler, James D. Roth, Robert J. Gau, Dennis L. Murray

**Affiliations:** ^1^ Department of Biological SciencesUniversity of ManitobaWinnipegManitobaCanada; ^2^ Department of Environment & Natural ResourcesGovernment of the Northwest TerritoriesYellowknifeNorthwest TerritoriesCanada; ^3^ Department of BiologyTrent UniversityPeterboroughOntarioCanada

**Keywords:** Age, intrapopulation variation, niche expansion, population cycles, resource limitation

## Abstract

Population dynamics of specialist carnivores are closely linked to prey availability, but the extent of variability in diet breadth of individual carnivores relative to natural variability in the abundance of their primary prey is not well understood. Canada lynx (*Lynx canadensis*) specialize on snowshoe hares (*Lepus americanus*) and exhibit cyclic fluctuations in abundance that lag 1–2 years behind those of snowshoe hares. Declining hare densities spur demographic changes in lynx, but it is unclear whether a corresponding increase in diet breadth occurs: (1) broadly across a lynx population; (2) only among individuals who are able to effectively switch to alternative prey; or (3) only among individuals who cannot capture sufficient primary prey. We measured stable isotope ratios of lynx muscle tissue spanning a cyclic increase and decline in hare density (1998–2001) in Fort Providence, NT, Canada. We found that lynx cohorts responded differently to hare population change, with yearling animals having broader diets at low hare densities, while adults and dependent juveniles maintained a constant diet through the initial decline in hare density. This result was consistent irrespective of lynx sex and indicates that yearling lynx likely are forced to adopt a broader diet when primary prey densities decline. Our results imply that select cohorts of specialist carnivores can exhibit high dietary plasticity in response to changes in primary prey abundance, prompting the need to determine whether increased diet breadth in young lynx is a successful strategy for surviving through periods of snowshoe hare scarcity. In this way, cohort‐specific niche expansion could strongly affect the dynamics of organisms exhibiting population cycles.

## Introduction

Food limitation is a critical environmental factor shaping the dynamics of consumers, and organisms may expand their dietary breadth to contend with limited food availability (Van Valen 1965). Yet, individuals vary in age, size, sex, and foraging experience, meaning that certain segments of the population are likely predisposed to lower success when acquiring limiting resources (Polis 1984; Kramer et al. 2015). This means that individuals with reduced ability to acquire scarce resources must use alternative prey, disperse, or die. Prey switching may enhance resilience of the population to environmental stressors by reducing intraspecific competition, mitigating consequences to productivity and thus may curb the rate of population decline. In this way, dietary plasticity may buffer fluctuations in population size by reducing mortality and enabling continued reproduction under marginal conditions; thus, it is essential to measure both population‐wide and demographic responses to limiting resources in order to understand the impacts of prey limitation on population dynamics (Wennersten and Forsman 2012).

Dietary niche expansion of consumer populations may be driven by increased variability within individual diets or by divergence of individual diets. First, if alternative prey are easy to catch relative to primary prey, then all members of a population may exhibit dietary plasticity when primary prey are scarce (Roughgarden 1972). On the other hand, if alternative prey is especially difficult for consumers to capture, then diet switching may be restricted to effective or experienced hunters (i.e., adults). For example, as recruitment of Eurasian perch (*Perca fluviatilis*) increases, adults are able to switch from feeding on benthic invertebrates to cannibalizing energy‐rich juveniles, but subadults maintain a diet of invertebrates (Persson et al. 2000). Alternatively, if prey switching occurs among individuals who are unable to effectively capture primary prey at low densities, then young, free‐living animals should have broader diets during primary prey shortages. Ungulates are the preferred prey of cougars (*Puma concolor*) and, during summer, ungulates comprise the largest proportion of cougar diet for all segments of the population. During winter, availability of ungulates diminishes and subadult diets expand to include a variety of nonungulate prey while adults maintain a considerable proportion of ungulate prey in their diet (Knopff et al. 2010).

**Figure 1:**
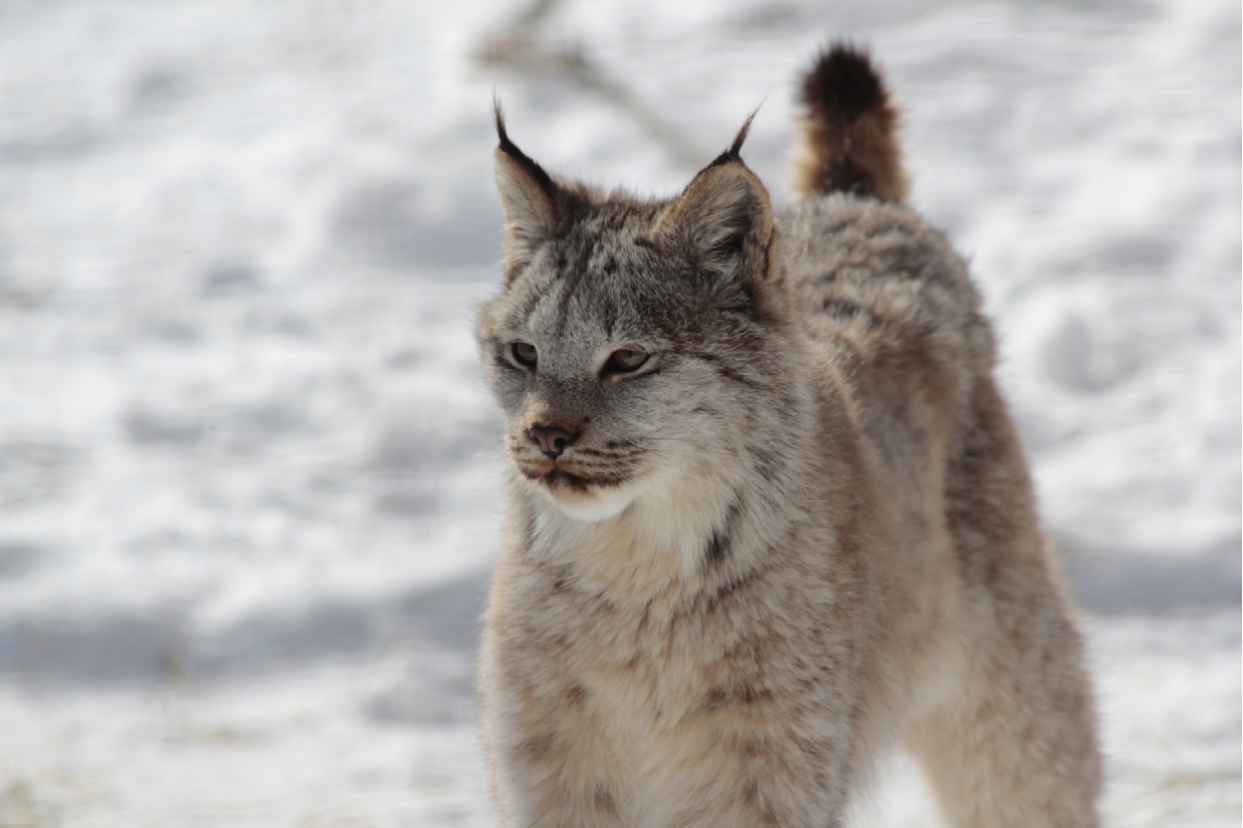
A yearling Canada lynx (*Lynx canadensis*). Photograph credit: C.M. Burstahler.

Canada lynx (*Lynx canadensis*) are specialist predators in a prey‐limited system, providing a useful model for examining effects of food limitation on diet breadth and its variability in a population (Fig. 1). Lynx in the core range exhibit population cycles following 1–2 years behind that of their preferred prey, snowshoe hare (*Lepus americanus*), which cycle in abundance every 9–10 years (Krebs et al. 2013). Lynx cycles have been attributed ultimately to changes in snowshoe hare density (Krebs et al. 2001) and proximately to changes in juvenile recruitment (Brand and Keith 1979; Mowat et al. 1996; Slough and Mowat 1996). Additionally, lynx can increase their use of alternative prey in their winter diets when hare densities decline (Brand et al. 1976; O'Donoghue et al. 1998a), meaning that the influence of primary prey can be dampened during periods of food shortage. During winter in the boreal forest, red squirrel (*Tamiasciurus hudsonicus*) comprise the greatest available alternative resource for lynx (Mowat et al. 2000), and ruffed grouse (*Bonasa umbellus*), small mammals (*Peromyscus* spp., *Microtus* spp.), and ungulate carrion also are consumed (Van Zyll de Jong 1966; Brand et al. 1976).

Alternative prey may be important to lynx populations during cyclic lows (O'Donoghue et al. 1998b; Roth et al. 2007), but it remains unclear whether variability in diet breadth is pervasive across a population, or whether certain individuals within select cohorts are more likely to switch prey. We examined lynx diet breadth variability among cohorts from a harvested population near Fort Providence, Northwest Territories, Canada. Our previous work (Roth et al. 2007) revealed that snowshoe hare are depleted in both *δ* ^13^C and *δ* ^15^N compared to alternative prey species, facilitating inference of lynx diet from stable isotope ratios. Because hare densities varied threefold during our study, we predicted that diet breadth would vary through time, with stable isotope ratios from tissue samples being more characteristic of those indicative of alternative prey, during periods of hare paucity (greater *δ* ^13^C and/or *δ* ^15^N). In following with the three alternative mechanisms of intrapopulation niche expansion outlined above, we predicted that lynx niche expansion during periods of low hare abundance would occur either: (1) across the population (if alternative prey are profitable and easily captured); (2) only among adults (if alternative prey are difficult to capture); or (3) only among independent subadults (if alternative prey are not profitable and represent a food of last resort for individuals that are unable to capture sufficient primary prey when at low densities).

## Methods

To examine annual variability in lynx diets, we measured stable isotope ratios of lynx harvested by fur trappers within 100 km of Fort Providence, Northwest Territories, Canada (61.35 N, 117.65 W). The study region is within the southern portion of the Taiga Plains ecological region (Ecosystem Classification Group 2007), and is mostly flat with large lakes and stands of coniferous and mixed boreal forest. Winters have mean temperature of −19°C, while summers have a mean temperature of 13°C; annual precipitation ranges from 350 to 450 mm (Poole 1989). Throughout the territory, lynx and snowshoe hare populations undergo periodic 10‐year cycles in abundance (Murray et al. 2008; Krebs et al. 2013).

We collected muscle samples from lynx harvested from November through March in four winters from 1997 to 1998 to 2000 to 2001 (hereafter referred to as winters 1998 through 2001). Lynx age was determined by counting cementum annuli of canine teeth (Matson's Laboratory LLC, Milltown, MT). Lynx were grouped into four age classes for analysis: juveniles (<1 year old), yearlings (1 year old), subadults (2 years old), and adults (≥3 years old). We considered subadults separately because the age of first reproduction increases when snowshoe hare are scarce (Parker et al. 1983; Slough and Mowat 1996). Hair of snowshoe hare and red squirrel was collected in 2001 to provide estimates of prey stable isotope ratios in the study area (see Roth et al. 2007).

Harvested lynx samples underestimate the number of kits (36%) and individuals ≥2 years old (4%) while overestimating the number of yearlings (40%) (Slough and Mowat 1996). We applied this correction factor to our harvest sample to provide a better estimate of population age structure. We calculated annual recruitment as the ratio of juveniles to adults (≥3 years old), based on population estimates corrected for harvest bias.

Snowshoe hare abundance was estimated each year using fecal pellet counts (Krebs et al. 2001) from seven sites throughout the Northwest Territories. At each site 4–6 transects with 70–100 plots, each (5.08 × 305 cm, spaced at 25 m intervals; see Poole 1989) were surveyed. Hare density was estimated from pellet counts using a regression equation developed for Yukon Territory (Krebs et al. 2001). Estimates reflect hare density of the previous year, biased heavily toward winter hare abundance (Murray et al. 2005); thus, pellet counts from June 1998 reflect hares available for lynx trapped in winter 1997–1998.

To prepare samples for stable isotope analysis, muscle tissue was freeze dried and powdered with a mortar and pestle. As carbon stable isotope ratios of lipids differ significantly from that of other compounds (DeNiro and Epstein 1977), lipids were removed using a Soxhlet apparatus with petroleum ether for over 8 h, and then, samples were dried in an oven to eliminate the solvent. Hair tissue of prey was washed with soap and water, thoroughly rinsed, dried, homogenized, and wrapped in tin capsules. Sample stable isotope ratios (*δ* ^13^C and *δ* ^15^N) were measured on a continuous flow isotope ratio mass spectrometer at the University of Central Florida. Stable isotope ratios are expressed using conventional delta notation (Ben‐David and Flaherty 2012). As muscle tissue has a turnover rate of approximately 1–3 months (Boecklen et al. 2011), our samples reflect an average of winter diet and coincide with the temporal period of prey availability reflected in snowshoe hare pellet counts. Both snowshoe hare and red squirrel molt in autumn, so hair stable isotope ratios reflect prey during October–November of 2000 (coincident with lynx winter diet 2001).

### Data analysis

Snowshoe hare availability is an important predictor of lynx numerical and behavioral responses (O'Donoghue et al. 1997, 1998a); however, dietary niche expansion is expected at the onset of snowshoe hare decline when predator density is high and access to prey is diminishing. This critical point of predicted dietary niche dynamics is not likely to coincide with the lowest hare density value but rather follow a time lag, and thus, a linear model of diet and hare density was not considered appropriate. Further obscuring the story, measures of snowshoe hare density in Fort Providence and territory‐wide trends were incongruous, leading us to seek additional proxy factors to define the quality of environmental conditions experienced by the lynx population. It is well established that lynx recruitment responds strongly to prey availability and is more reliable than other measures of productivity such as placental scars or corpora lutea (Mowat et al. 1996). We characterized each year of study dichotomously as marginal or good by first considering local snowshoe hare density, second considering local recruitment of lynx, and finally confirming our designation by referring to mean hare density and lynx harvest for the Territory. We consider these as indicators of environmental conditions experienced by lynx and relevant at both broad and local spatial scales.

Population‐level diet responses are reflected in mean stable isotope ratios. Together, *δ* ^13^C and *δ* ^15^N provide a two‐dimensional measure of diet and thus are ideally modeled together using MANOVA; however, lack of homogeneity of variance–covariance and weak linear association between response variables raised concerns against use of MANOVA for these data. Linear mixed models allow for unequal variance structures but are not yet accessible for multiple response variables. As such, we considered *δ* ^13^C and *δ* ^15^N separately for population‐level diet responses. Note that hypotheses regarding each remain the same: both are predicted to increase during marginal conditions as use of alternative prey increases.

The influence of environmental condition, age class, and sex on mean lynx stable isotope ratios was evaluated using standard model selection procedures (Burnham and Anderson 2002) from a set of a priori linear mixed models fit by maximum likelihood. Fixed effects included the interaction of environmental condition (*EC*) and age class (*A*), as well as main effects of *EC*,* A,* and sex (*S*). Red squirrel stable isotope ratios are variable between seasons, years, and among individuals depending on conifer masting events, composition of food caches, and use of animal protein (Roth et al. 2007; appendix D). Red squirrels comprise the greatest available alternative resource to lynx during winter in northern latitudes (Mowat et al. 2000) and could strongly influence stable isotope ratios measured in lynx. As such, year of collection (*Y*) was included in all models as a random effect to account for unmeasured variation in prey community stable isotope ratios. Separate intercepts were fit for each year, but slopes of parameters were held constant. Raw values of *δ* ^13^C and *δ* ^15^N were analyzed as no transformations improved model fit. A null model was included to provide a baseline comparator for other models in the suite. The difference between a model's AIC value and the minimum AIC of the model (ΔAIC) provides a measure of Kulback–Leibler information loss relative to competing models and thus a measure of each model's plausibility relative to others in the set (Burnham and Anderson 2002, p. 70). Models with ΔAIC smaller than two were considered competitive, unless they differed by one parameter with little change in maximized log‐likelihood (Burnham and Anderson 2002, p. 131; Arnold 2010). The variance explained by each model was summarized as the marginal and conditional *R* ^2^, where marginal *R* ^2^ considers variance explained by fixed effects only while conditional *R* ^2^ reflects variance explained by both random and fixed effects (Nakagawa and Schielzeth 2013). Coefficients for all models were then recalculated using restricted maximum likelihood (REML) to improve estimation of random effects and balance unequal variance among predictors (Bolker et al. 2009). The relative importance of predictor variables to each of *δ* ^13^C and *δ* ^15^N was measured by summing the model weights (*w*_*i*_) of all models in which a predictor appears.

Individual‐level responses are reflected in the variability among individual stable isotope ratios, or diet breadth of a demographic group. We examined changes in diet breadth of cohorts based on the dispersion of stable isotope ratios in two‐dimensional space (i.e., the biplot of *δ* ^13^C and *δ* ^15^N values; Bearhop et al. 2004). The standard ellipse area (SEA) characterizes spatial variability in the stable isotope data as a two‐dimensional standard deviation, governed by the covariance matrix between *δ* ^13^C and *δ* ^15^N values and containing approximately 40% of the data (Jackson et al. 2011; see also Batschelet 1981). More diverse individual diets within a demographic group are characterized by greater spread in stable isotope ratios and thus a larger standard ellipse. We used the SEA_c_ metric, which improves estimation for small sample sizes (Jackson et al. 2011). Our large annual sample permitted partitioning of the data into age classes without compromising the precision of our SEA_c_ estimates.

We used a linear mixed‐effects model based on a split plot design to examine differences in SEA_c_ associated with environmental condition (between‐plot factor) and age class (within‐plot factor), including interactions between environmental condition and age class. Intercepts were allowed to vary by year of collection (random effect) to control for potential differences of prey community stable isotope ratios between winters. In its classical form, the split plot design assumes a single datum per cell and thus is appropriate for the single SEA_c_ estimate per grouping per year. We used ΔAIC to assess split plot model fit in comparison with simpler variations of the model, including a null model that considered only the random intercepts for year of collection. Explained variance was summarized as the marginal and conditional *R* ^2^. Confidence intervals for parameter estimates of the selected model were calculated using a basic bootstrap of 999 simulations (Thai et al. 2013). All analyses were conducted using the “lme4” (Bates et al. 2015) and “siar” (Parnell and Jackson 2013) package in R (R Core Team 2014).

## Results

The study period was characterized by variable snowshoe hare densities and lynx recruitment rates in Fort Providence (Table 1). Territory‐wide mean hare densities and total lynx harvest suggest a relatively low peak abundance for both lynx and hares, but estimates are highly variable across the landscape (Table 1). Good years include data collected winter of 1999 and 2000, represented by higher snowshoe hare densities (mean local hare densities: 0.53 and 0.90 hares/ha, respectively) and high lynx recruitment (mean recruitment rate: 1.58 and 1.87 juveniles/adult, respectively). Marginal years include data collected in 1998 and 2001, representing low hare densities (mean local hare densities: 0.45 and 0.62 hares/ha, respectively) and minimal recruitment (mean recruitment rate: 0.62 and 0.40 juveniles/adult, respectively).

**Table 1:** Summary of factors considered at local (Fort Providence, NT) and broad (territory‐wide) spatial scales for designation of environmental condition category (*EC*: G = good, M = marginal). Estimated age class structure of the study population is corrected for harvest bias based on Slough and Mowat (1996). Recruitment was calculated from age structure data corrected for harvest bias. The total number of lynx pelts harvested per winter in the Northwest Territories from 1997 to 1998 through 2000 to 2001 and the mean (and SD) estimated density of snowshoe hare from seven sites across the Northwest Territories from June 1998 to 2001 indicate broad scale environmental conditions

Year	*n*	Local measures						Territory‐wide measures		
		Juvenile (0 years)	Yearling (1 year)	Subadult (2 years)	Adult (3+ years)	Lynx recruitment (juv./adult)	Snowshoe hare density (hares/ha)	Lynx harvest (# pelts)	Snowshoe hare density (hares/ha)	*EC*
1998	112	0.13	0.33	0.33	0.21	0.62	0.04	709	0.09 (0.27)	M
1999	199	0.35	0.32	0.11	0.22	1.58	0.14	1491	1.12 (1.65)	G
2000	93	0.33	0.34	0.15	0.18	1.87	0.49	1330	1.24 (0.67)	G
2001	165	0.07	0.38	0.37	0.18	0.40	0.23	711	0.78 (0.55)	M

We collected 505 lynx carcasses comprising 112 lynx in 1998, 199 lynx in 1999, 93 lynx in 2000, and 165 lynx in 2001. Lynx age, estimated from tooth cementum annuli, ranged from juveniles (<1 year) to 12 years old, and the sample included 14.0% juveniles, 49.4% yearlings, 19.8% subadults, and 16.7% adults overall. The sex ratio of sampled lynx was 58.4% male and the age distribution of the population (corrected for harvest bias) fluctuated between years (Table 1).

Stable isotope ratios of snowshoe hares (*n* = 6, *δ* ^13^C = −26.28 ± 0.93‰ SD, *δ* ^15^N = 3.25 ± 1.44‰ SD) and red squirrels (*n* = 6, *δ* ^13^C = −20.70 ± 0.53‰ SD, *δ* ^15^N = 9.91 ± 3.21‰ SD) collected in 2001 were distinct and encompassed the range in stable isotope ratios observed for lynx.

Model selection suggests that all hypothesized parameters were potentially important predictors of mean *δ* ^15^N, but had little effect on mean *δ* ^13^C (Table 2). Supported models of mean *δ* ^15^N suggest that an interaction between environmental condition and age class, and/or the main effects of *EC* and *A,* influenced lynx population stable nitrogen ratios. While both Model 1 and the Global model are within two ΔAIC values, the log‐likelihoods are essentially equivalent indicating that the addition of sex does little to improve predictive performance of the model. Random intercepts for Model 1 were 1998 = 4.33, 1999 = 4.57, 2000 = 4.54, and 2001 = 4.77. Note that variance in *δ* ^15^N explained by fixed effects is considerably better than models of *δ* ^13^C and that inclusion of variance explained by the random effect of year improves the explained variance only slightly. Cumulative Akaike weights (CW) suggest that environmental condition (CW = 0.90), age class (CW = 0.73), and their interaction (CW = 0.58) were important predictors of *δ* ^15^N, but sex was of little importance (CW = 0.29). Coefficients for all models are presented in Table 3.

**Table 2:** Linear mixed models compared to identify predictors of mean Canada lynx population diet reflected in stable isotope ratios for *δ* ^13^C and *δ* ^15^N (*n* = 505). Hypotheses included an interaction between environmental condition (*EC*) and age class (*A*)*,* their main effects, and sex (*S*) as fixed effects. Random intercepts were fit for each year of collection (*Y*) to account for annual variation in stable isotope ratios of the prey community. For each model: *K* refers to the number of parameters; log‐likelihood (*L*) indicates the probability of observed values; ΔAIC is the difference between each model and the minimum AIC; model weight (*w*_*i*_) is the relative likelihood of each model (exp(−0.5 ΔAIC) divided by the sum of relative likelihoods of all models in the set; $R_{m}^{2}$ represents the marginal variance explained (fixed effects only); and $R_{c}^{2}$ represents the conditional variance explained by both fixed and random effects

Model no.	Model	*K*	*δ* ^13^C					*δ* ^15^N				
			*L*	ΔAIC	*w*_*i*_	$R_{m}^{2}$	$R_{c}^{2}$	*L*	ΔAIC	*w*_*i*_	$R_{m}^{2}$	$R_{c}^{2}$
Global	*EC*:*A* + *EC* + *A* + *S* + (1|*Y*)	11	−196.10	10.57	0.00	0.03	0.18	−**545.47**	**1.72**	**0.17**	0.12	0.16
1	*EC*:*A* + *EC* + *A* + (1|*Y*)	10	−196.86	10.10	0.00	0.02	0.18	−**545.62**	**0.00**	**0.41**	0.12	0.16
2	*EC* + *A* + *S* + (1|*Y*)	8	−196.32	5.01	0.02	0.03	0.18	−550.18	5.12	0.03	0.11	0.15
3	*EC* + *A* + (1|*Y*)	7	−197.05	4.47	0.03	0.02	0.18	−550.24	3.24	0.08	0.11	0.15
4	*EC* + *S* + (1|*Y*)	5	−**197.72**	**1.82**	**0.12**	0.02	0.17	−552.59	3.95	0.06	0.10	0.14
5	*A* + *S* + (1|*Y*)	7	−196.48	3.34	0.06	0.01	0.17	−552.23	7.23	0.01	0.01	0.14
6	*EC* + (1|*Y*)	4	−**198.60**	**1.57**	**0.14**	0.02	0.17	−**552.61**	**2.00**	**0.15**	0.10	0.14
7	*A* + (1|*Y*)	6	−197.21	2.79	0.07	0.01	0.17	−552.30	5.36	0.03	0.01	0.14
8	*S* + (1|*Y*)	4	−**197.94**	**0.26**	**0.26**	0.00	0.17	−554.74	6.24	0.02	0.00	0.14
Null	1 + (1|*Y*)	3	−**198.81**	**0.00**	**0.30**	0.00	0.17	−554.76	4.30	0.05	0.00	0.14

Models with ΔAIC < 2 were supported by the data and are highlighted in bold.

**Table 3:** Parameter estimates of fixed effects for linear mixed models of mean *δ* ^13^C (a) and *δ* ^15^N (b) of Fort Providence lynx. Models are presented in descending order from highest to lowest model weight from model selection (see Table 2). Deflections for environmental condition are from good to marginal in all cases. Deflections for age class are from juveniles to the age class in parentheses: yearlings (*y*), subadults (*s*), and adults (*a*). Deflections for sex are from male to female

Model no.	Model	Intercept	*EC*:* A*(*y*)	*EC*:* A*(*s*)	*EC*:* A*(*a*)	*EC*	*A* (*y*)	*A* (*s*)	*A* (*a*)	*S*
(a) *δ* ^13^C										
Null	1 + (1|*Y*)	−24.90								
8	*S *+ (1|*Y*)	−24.92								0.04
6	*EC* + (1|*Y*)	−24.90				−0.07				
4	*EC* + *S *+ (1|*Y*)	−24.92				−0.07				0.04
7	*A *+ (1|*Y*)	−24.84					−0.07	−0.09	−0.05	
5	*A *+* S *+ (1|*Y)*	−24.86					−0.07	−0.09	−0.05	0.04
3	*EC* + *A *+ (1|*Y*)	−24.84				−0.06	−0.07	−0.09	−0.05	
2	*EC* +* A *+ *S *+ (1|*Y*)	−24.86				−0.06	−0.07	−0.09	−0.05	0.04
1	*EC*:*A *+ *EC *+ *A *+ (1|*Y*)	−24.83	−0.03	−0.05	−0.04	−0.03	−0.09	−0.10	−0.06	
Global	*EC*:*A *+ *EC *+ *A *+* S *+ (1|*Y*)	−24.84	−0.03	−0.06	−0.04	−0.03	−0.08	−0.09	−0.06	0.04
(b) *δ* ^15^N										
1	*EC*:*A *+ *EC* + *A *+ (1|*Y*)	4.55	−0.11	0.02	−0.41	0.45	0.13	0.05	0.00	
Global	*EC*:*A *+ *EC* +* A *+* S *+ (1|*Y*)	4.54	−0.11	0.02	−0.42	0.45	0.13	0.06	−0.01	0.03
6	*EC* + (1|*Y*)	4.63				0.34				
3	*EC* + *A *+ (1|*Y*)	4.50				0.32	0.18	0.16	0.05	
4	*EC* + *S *+ (1|*Y*)	4.62				0.34				0.01
Null	1 + (1|*Y*)	4.63								
2	*EC* +* A *+ *S *+ (1|*Y*)	4.49				0.32	0.18	0.16	0.05	0.02
7	*A *+ (1|*Y*)	4.50					0.19	0.17	0.06	
8	*S *+ (1|*Y*)	4.62								0.01
5	*A *+* S *+ (1|*Y)*	4.48					0.19	0.17	0.06	0.02

In contrast, supported models for *δ* ^13^C included the null, suggesting that hypothesized predictors had no measurable effect on lynx stable carbon ratios. Indeed, the variance explained by fixed effects alone ($R_{m}^{2}$) was almost negligible for *δ* ^13^C while the variance explained by year of collection ($R_{c}^{2}$) was considerably higher. Fit of the global model for *δ* ^13^C satisfied the assumptions of normality and homogeneity of variance. Random intercepts were 1998 = −24.76, 1999 = −24.95, 2000 = −25.75, and 2001 = −25.14. Cumulative AIC weights (CW) for predictors of *δ* ^13^C were EC:*A *=* *0.01, EC = 0.31, *A* = 0.19, and *S* = 0.46.

**Figure 2:**
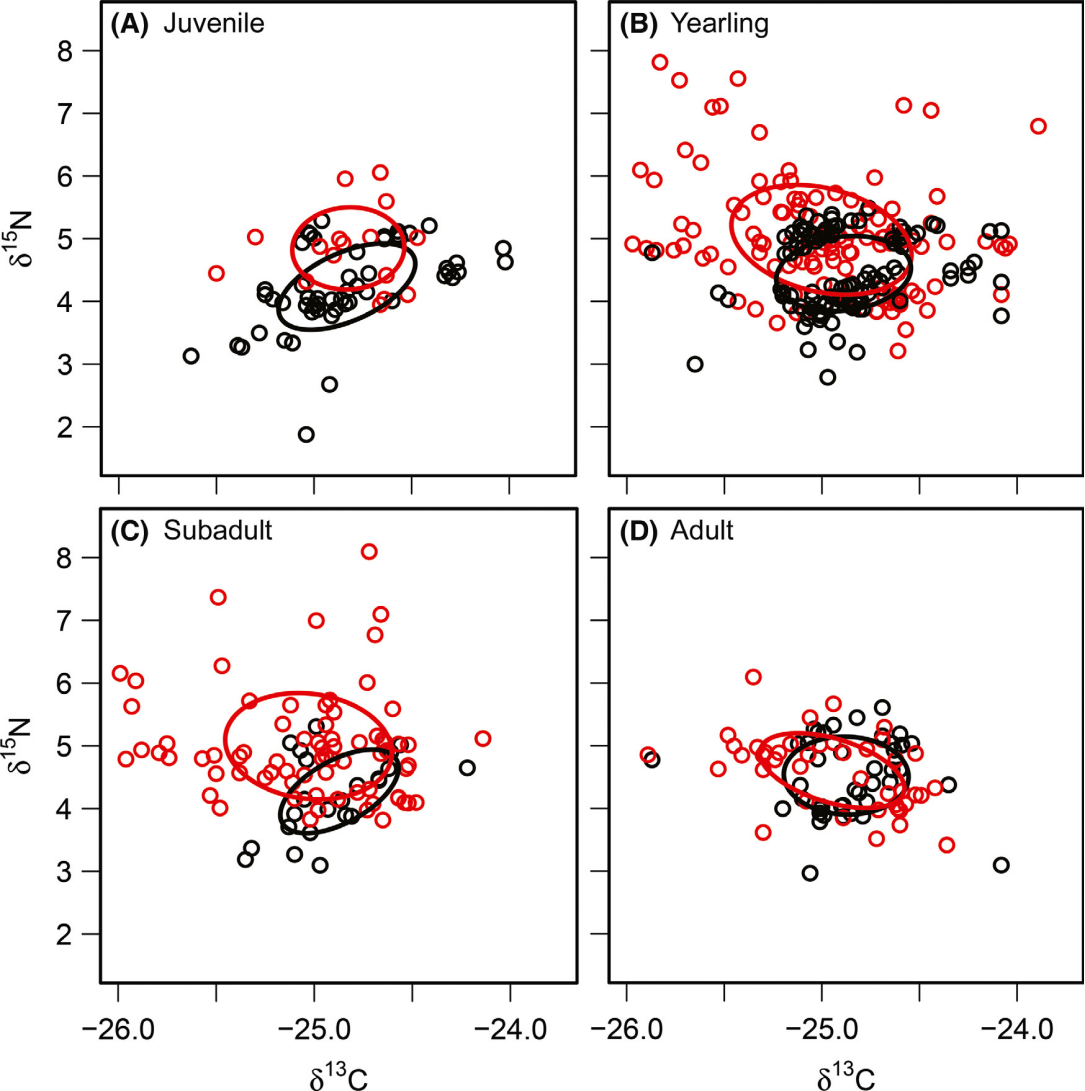
Age‐structured differences in isotopic niche breadth (SEA) of lynx during marginal environmental conditions when snowshoe hare densities and lynx recruitment were low (red), and during good environmental conditions when snowshoe hare densities and lynx recruitment were high (black) for A) juvenile lynx, B) yearling lynx, C) subadult lynx, and D) adult lynx. SEA is the standard ellipse area.

**Figure 3:**
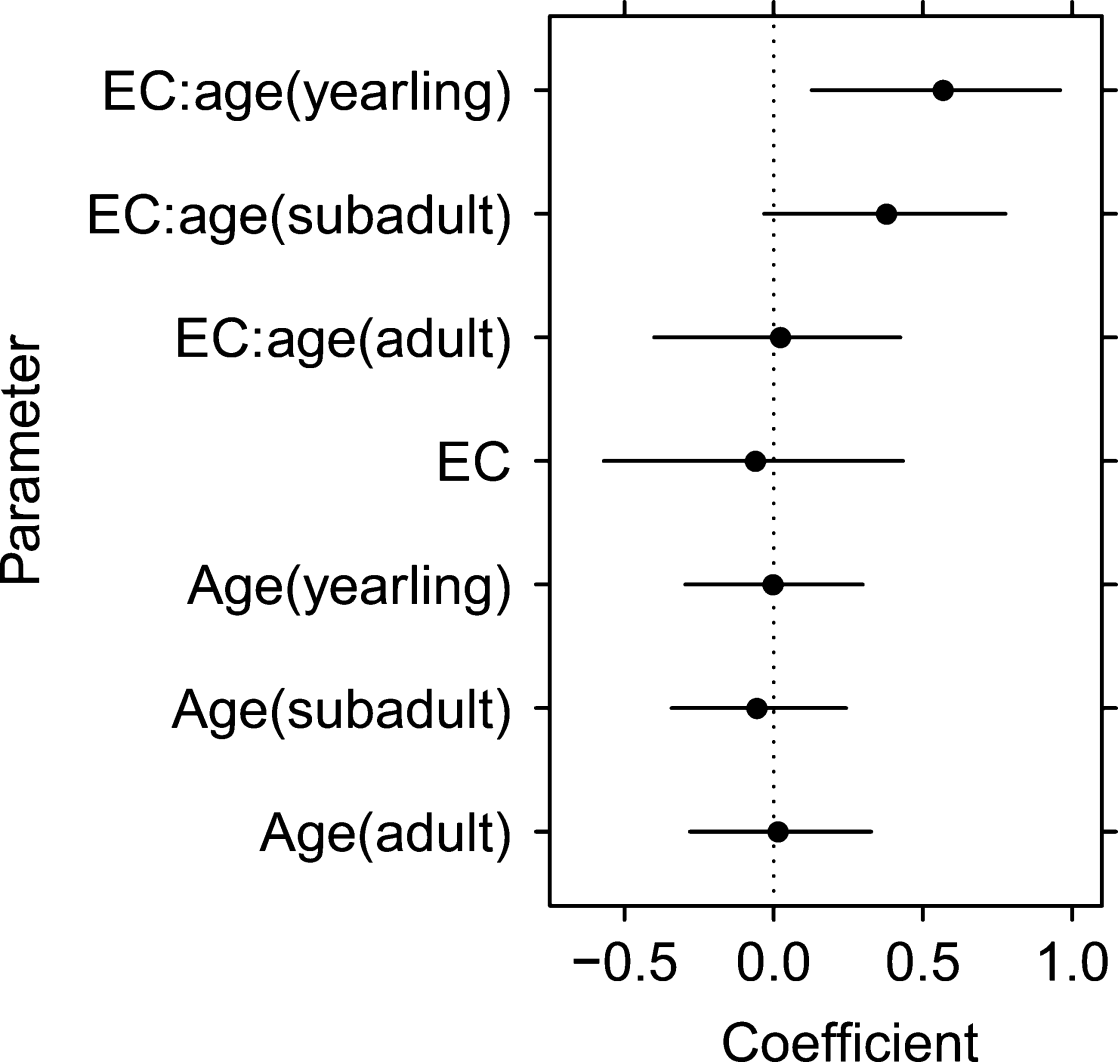
Parameter estimates of a split plot linear mixed‐effects model describing variation in standard ellipse area (SEAc) estimates of lynx diet breadth by environmental condition, age class, an interaction of environmental condition and age class, and random intercepts for year of collection. Deflections for environmental condition are from good to marginal in all cases. Deflections for age class are from juveniles to the age class in parentheses. Error bars represent the 95% confidence interval of a basic bootstrap on parameter estimates.

Stable isotope ratios and SEA_c_ estimates of lynx grouped by environmental condition and age class are presented in Figure 2. Diet breadth estimates (SEA_c_) of cohorts during good years were juveniles = 0.64‰^2^, yearlings = 0.64‰^2^, subadults = 0.52‰^2^, and adults = 0.62‰^2^. Diet breadth estimates (SEA_c_) of cohorts during marginal years were juveniles = 0.59‰^2^, yearlings = 1.22‰^2^, subadults = 1.12‰^2^, and adults = 0.61‰^2^. The global split plot model of diet breadth fit better than simpler variations (Table 4). Diet breadth of yearlings expanded when environmental condition declined, but juveniles, subadults, and adults maintained largely similar diets across environmental conditions (Fig. 3). The coefficient for subadult niche expansion under marginal environmental conditions may suggest a tendency toward niche expansion, but confidence intervals of our model overlap zero (Fig. 3). Random intercepts for the global model were 1998 = 0.60, 1999 = 0.50, 2000 = 0.72, and 2001 = 0.37. Model fit improved considerably with addition of random effects ($R_{m}^{2}$ = 0.37 and $R_{c}^{2}$ = 0.77). Visual inspection of fitted values and residuals indicated compliance with assumptions of normality and homoscedasticity.

**Table 4:** Model selection results from an analysis of lynx diet breadth (estimated as standard ellipse area, corrected for small sample size (SEAc); *n* = 16) as predicted by environmental condition (EC), age class (*A*), the interaction between environmental condition and age class (EC:*A*), and random intercepts for year of collection (*Y*). For each model: *K* refers to the number of parameters; log‐likelihood indicates the probability of observed values; ΔAIC is the difference between each model and the best fit model; model weight is the relative likelihood of each candidate model (exp(−0.5 ΔAIC) divided by the sum of relative likelihoods of all models in the set; $R_{m}^{2}$ represents the marginal variance explained (fixed effects only); and $R_{c}^{2}$ represents the conditional variance explained by both fixed and random effects

Model no.	Model	*K*	*L*	ΔAIC	*w*_*i*_	$R_{m}^{2}$	$R_{c}^{2}$
1	SEA_c_ = *EC*:*A *+* EC* +* A *+ (1|*Y*)	10	8.9	0.0	0.9	0.5	0.8
2	SEA_c_ = *EC *+ (1|*Y*)	4	0.4	4.9	0.1	0.1	0.3
3	SEA_c_ = *A *+ (1|*Y*)	6	2.4	5.0	0.1	0.2	0.5
Null	SEA_c_ = 1 + (1|*Y*)	3	−0.2	6.4	0	0	0.3
4	SEA_c_ = *EC *+* A *+ (1|*Y*)	7	3.0	8.0	0	0.3	0.5

## Discussion

Our results indicate that at the onset of a cyclic decline in snowshoe hare abundance, it is those segments of the lynx population that were unable to effectively capture primary prey that drove niche expansion. In our study, yearling lynx increased their use of alternative prey when faced with marginal environmental conditions; however, adults and their dependent juveniles maintained a narrow diet of snowshoe hare throughout the initial period of declining hare abundance. As our study only captures the beginning of the cyclic decline we cannot speak to dietary plasticity of the population during cyclic lows when recruitment has collapsed and adults predominate. Indeed, O'Donoghue et al. (1998b) describe the greatest dietary niche expansion 3 years following peak hare density in Kluane, YT suggesting that adults do, ultimately, use alternative prey, becoming sufficiently skilled at hunting squirrels that they do not immediately resume hunting hares at the onset of increasing hare densities. Thus, it is possible that the yearling lynx forced to seek out alternative prey during the initial decline of snowshoe hare abundance develop critical hunting skills that increase survivorship throughout nadirs of the cycle.

The age class effect demonstrated herein suggests that the facultative nature of lynx specialization is more nuanced than previously described (O'Donoghue et al. 1998b; Roth et al. 2007; Murray et al. 2008). If prey switching incurred immediate benefits to lynx, then all segments should be equally likely to expand their diet breadth in immediate response to their environment; yet, our data only provide evidence for those animals that are naïve hunters spending their first winters as independent, free‐ranging individuals. The observed changes in diet breadth among newly independent lynx may reflect hunting inexperience (Polis 1984), limited access to good hare habitat, or responses to intraspecific competition (Svanbäck and Bolnick 2007). Collectively, our data suggest that use of alternative prey is not easy (otherwise we would expect a population‐wide response), nor do alternative prey present a more profitable food source (in which case more experienced, adult lynx would use them), but rather that the switch to alternative prey is likely a last resort for select individuals, following limitation of their primary prey. Juvenile lynx forage in mother‐kit groups during their first winter (McCord and Cardoza 1982; Mowat et al. 1996), and so it is not surprising that juvenile diet coincided with that of adults. Subadult lynx show a tendency toward niche expansion which may reflect differences in speed of acquisition of hunting skills among developing lynx.

Prey switching is a form of behavioral plasticity that allows rapid responses to environmental change, yet it is not always clear whether switching is beneficial or not (Tuomainen and Candolin 2011). One to 2 years following snowshoe hare decline, lynx populations experience increased mortality largely due to human trapping, intra‐ and interspecific strife, and starvation (Slough and Mowat 1996; O'Donoghue et al. 1997). Assuming that use of alternative prey is advantageous to young lynx, the tendency toward more variable diets may contribute to the resilience of certain individuals to survive through nadirs of the snowshoe hare cycle. While recruitment and mortality remain the prime drivers of the lynx cycle, it is possible that niche expansion of younger age classes may curb the rate of population decline through enhanced survivorship of some individuals (Agashe and Bolnick 2012). Thus, there remains a need to determine more fully if use of alternative prey by young lynx is a successful strategy for surviving through periods of hare scarcity.

A number of factors distinguish our study period as an unconventionally low fluctuation in the history of lynx‐hare population cycles in the Northwest Territories (Fig. 1; Elton and Nicholson 1942; see Mackenzie Basin in fig. 8, p. 232). Peak snowshoe hare density was roughly fivefold less than historic peaks in snowshoe hare abundance for Fort Providence (7–9 hares/ha, Poole 1994; 5.3–6.2 hares/ha, Poole 1997). Previous age class estimates from harvested lynx in Fort Providence (1989–1993) found juveniles to range from zero during periods of low hare density to 49% when hares were abundant (Poole 1994); similar figures were reported in other parts of the lynx range (Brand and Keith 1979; Parker et al. 1983; Slough and Mowat 1996). In contrast, our study comprised a small proportion of juveniles (14%) over the 4‐year period and a lower peak recruitment (1.9 juveniles/adult).

Environmental condition was a good predictor of lynx stable isotope ratios, although models of *δ* ^13^C and *δ* ^15^N did not fully agree (Table 2). Our best explanation for this disjunction is that stable isotope data are inherently noisy because so many environmental and physiological factors affect them. We speculate that the disagreement between stable carbon and stable nitrogen models may be explained by noise generated via several competing inputs to the data set. First, our sample represents harvested lynx only, which means that we lack data for lynx that died from starvation and it is probable that a portion of our sample represents dispersers moving through the area from another region with a different prey base. Prey stable isotope ratios vary geographically (Roth et al. 2007), and thus, lynx originating from other regions would reflect these differences, adding noise to the system. Second, red squirrel stable isotope ratios vary temporally in both carbon and nitrogen (Roth et al. 2007), further contributing to noise. Finally, it is possible that some lynx were using a different species of alternative prey that does not differ greatly from hares in *δ* ^13^C, and thus, we see a clear signal for diet switching in *δ* ^15^N only. Because of these confounding influences we chose to analyze variability of lynx stable isotope ratios in addition to mean diet explained by the linear mixed models, to clarify differences in diet breadth in the absence of adequate prey stable isotope data.

The lynx of Fort Providence, NT appear to be highly sensitive to even minor changes in snowshoe hare densities, demonstrating measurable responses in both resource use and reproductive output. The cumulative evidence suggests that greater availability of snowshoe hare coincides with greater consumption of hares by all segments of the population and enables increased recruitment to the population. At the population level, our data support a trade‐off of investing in reproduction when resources are abundant and prioritizing survival when resources are limiting. Demographically, our data demonstrate an age class effect whereby young, naïve individuals exploit alternative resources to contend with limited availability of snowshoe hare, while adults and their kits are able to maintain constant diets during initial hare decline. The age class effect provides a more precise definition for the degree of dietary flexibility exhibited by a specialist consumer, and implies a link between diet, survivorship, and population dynamics.

Lynx at the southern periphery of their range have demonstrated population declines and have been federally protected as a Threatened species in the contiguous United States (USFWS 2000; Murray et al. 2008). The southern periphery is characterized by lower snowshoe hare densities and dampened population cycles (Hodges 2000; Murray 2000). Intrapopulation variation can potentiate larger range size and reduce extinction risk (Wennersten and Forsman 2012), and thus, the propensity of young lynx to use alternative prey may be a pivotal characteristic to maintain lynx populations in regions with chronically low snowshoe hare abundance. Future studies addressing such complexities will be critical to conservation and harvest management planning for lynx as well as for other carnivores that also demonstrate spatiotemporal variability in prey choice and diet breadth.

## Data Accessibility

Archived data used in this manuscript will be made available through Dryad.

## Conflict of Interest

None declared.
